# Supplementary material for: Oxidative Stability and Pasting Properties of High-Moisture Japonica Brown Rice following Different Storage Temperatures and Its Cooked Brown Rice Flavor
Source: Foods. 2024 Feb 2;13(3):471. doi: 10.3390/foods13030471 (PMC10855601; doi:10.3390/foods13030471)
Supplement: Supplementary file 1 [file foods-13-00471-s001.zip › foods-2823938-supplementary.pdf]

Supporting Information

For

Oxidative Stability and Pasting Properties of High-moisture Japonica Brown Rice Following Different Storage Temperatures and Its Cooked Brown Rice Flavor

Lingyu Qu, Yan Zhao*, Yanfei Li, and Haoxin Lv

School of Food and Strategic Reserves, Henan University of Technology, Zhengzhou, 450000, PR China

Corresponding Author

* Prof. Yan Zhao (Y. Zhao).

Email: zhaoyan@haut.edu.cn

Supporting information Figure. S1

Figure. S1

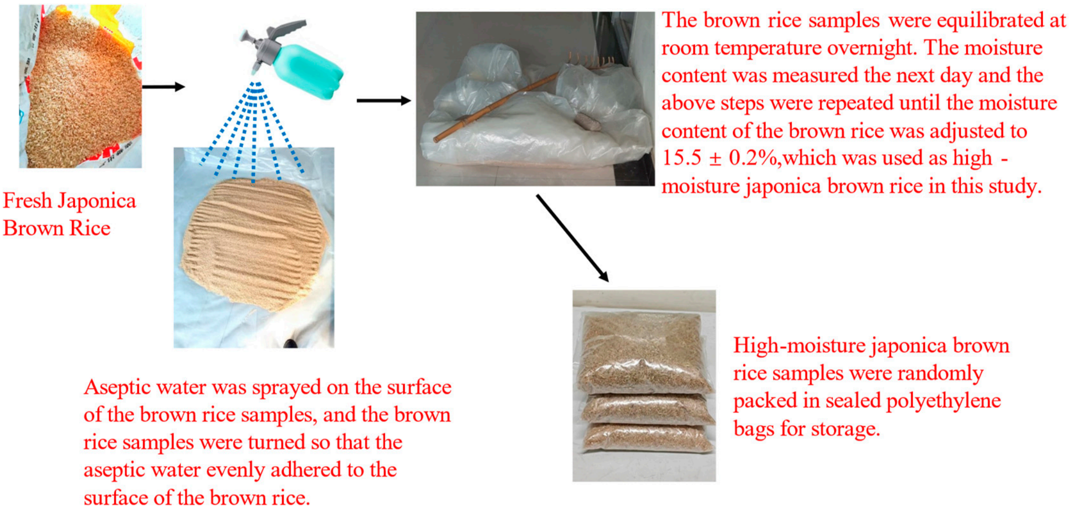

Figure. S1. Preparation of brown rice samples with high moisture content in the japonica variety

Figure. S2

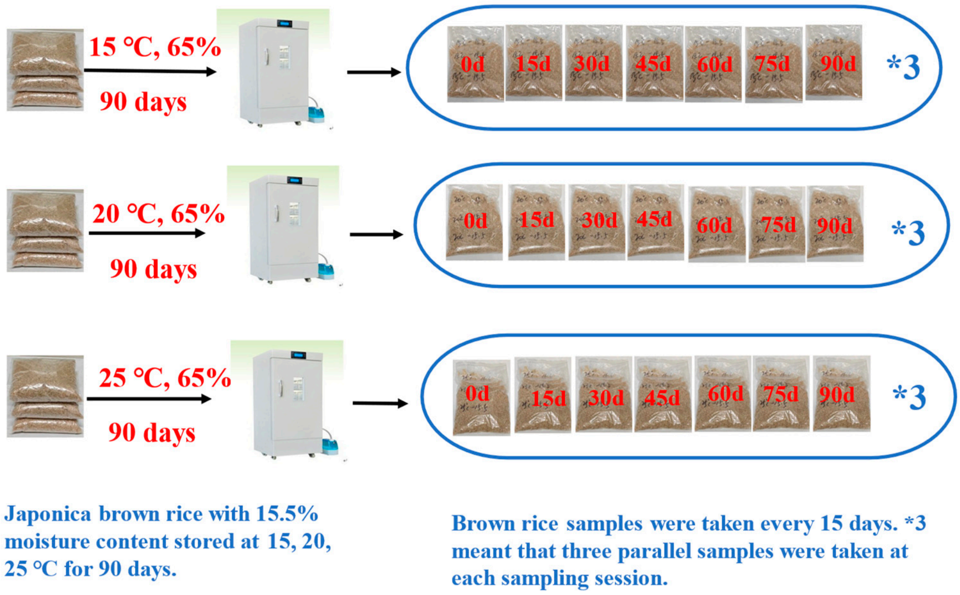

Figure. S2. Collection of brown rice samples with high moisture content in the japonica variety
